# The Endoscope, and Its Application to the Diagnosis and Treatment of Urinary Affections

**Published:** 1867-09

**Authors:** A. J. Desormeaux


(*Continued from page 400.*)

into the perineum hard nodosities, readily felt by the fingers. A No. 5 bougie could be readily passed, with a slight discharge of blood; it was kept *in situ* for about two hours, then, on being withdrawn, the patient urinated somewhat more readily.

*5th Jan.* A No. 7 bougie is readily introduced, but remains pinched by the coarctation; it was left about two hours. The urinary function is more easy.

*6th Jan.* The patient is fatigued; he has had no stools since his entrance into the hospital; a bottle of Seidlitz water was ordered. The result was, not large evacuations, slight chills, and pain in the loins. He remains in bed, and takes only a light soup.

*7th Jan.* Leave him quiet, and give an enema, as follows: ℞. Valerian, gram. 2 (grs. 80), quin. sulph. 1 gram. (grs. 40), laudanum, Rousseau's, 10 gtt., camphor, 2 gram. (80 grs.)

*8th Jan.* This morning, the patient seeming well, the endoscope was introduced, it showed a retraction of the canal of the normal color; the stylet was passed, the mucous membrane in front of the coarctation was perfectly healthy. He passed the day well. At 3½ P.M., he took an enema of quinine, after having first taken a simple one which brought away much fæcal matter; immediately he was seized with dizziness, palpitation, nausea, cold sweats, vertigo, and ringing in the ears. A few glasses of Seidlitz water and coffee without milk, and the sickness was allayed.

*9th.* This morning the patient does well; rest is given him. He is purged with a bottle of Seidlitz water, and takes an enema of 10 grs. (50 centigrammes) of the sulphate of quinine. In the evening he experienced the same symptoms as on the previous day, but less decided.

*11th and 12th.* The injections are repeated, and no return of the sickness; the febrile symptoms have not reappeared. A No. 4 bougie was introduced and left in for two hours. He experienced a pricking sensation in the canal, and some drops of blood came out with the first drops of urine after the withdrawal of the bougie. The flow is somewhat larger.

*13th.* This day was well passed; the enema was countermanded.

*14th.* The endoscope was again employed this morning. About 3 P.M, he had an intense chill, faintness, thirst, nausea, and pain in the kidneys; micturition was very painful, and for some hours left a condition of sickness.

*15th.* He was better. A bougie, No. 21, was introduced as far as the stricture and left for fifteen minutes, for the purpose of decreasing the sensibility of the canal and of dilating the coarctated spongy portion. The evening found him slightly fatigued, the bougie was reintroduced, and the injection discontinued.

*16th.* Same condition. Seidlitz water.

*30th.* Every day a large bougie has been introduced as far as the stricture; the general health is good. The endoscope is now applied, and a superior incision made the length and thickness of the coarctation. The discharge of blood is but slight. Immediately after, a No 20 bougie is introduced and fixed; the urine flows, the inconvenience to the patient is slight. In the evening he complains of sharp pains in the part operated upon, radiating towards the anus, but without either chills or fever. An enema of quinine is prescribed. The urine is freely passed, and there is a sero-purulent and somewhat sanguinolent discharge between the sound and meatus.

*Feb. 1st.* Condition satisfactory; sound withdrawn; bowels costive; tongue white; loss of appetite. Seidlitz water.

*2d.* Discharge diminished; still some pain in urinating, the flow being neither strong nor rapid, but better than before the operation. There is some suffering in the perineum and in the lower belly. Cataplasms.

*5th.* A No. 22 bougie introduced and left in place for ten minutes.

*6th.* Bougie No. 23. In the evening, chills and discomfort.

*7th.* Pass a smaller bougie and withdraw it immediately. During the day, he experiences slight chills, indisposition, a natural condition, and considerable pain in the parts cut. Injection of quinine.

*11th.* Each day, a metallic bougie is introduced and at once withdrawn; the urine passes well, but he is fatigued; his eyes are encircled, and almost every day he experiences slight chills.

*20th.* Sounds of Béniqué, Nos. 30 to 37, successively introduced without difficulty or pain. The condition of the patient, and by his own acknowledgment, is much improved.

*22d.* Bougies 35 to 41 sufficiently large. The patient complains of nothing, is up, eats heartily, urinates easily and without pain, and may be considered as cured.

Among the accidents which may be the consequence either of stricture or of traumatic lesion of the urethra, but few are more serious or more distressing than urinary fistula. To give their entire history would lead us from our legitimate subject; thus we must be content to confine ourselves to the results of endoscopic aid in their cure.

The usual treatment of urethral fistula consists in the employment of permanent sounds or repeated catheterism, after dilatation. By these means, it is proposed to prevent the flow of urine through the fistula, which most frequently cicatrizes when this secretion does not tend, by its passage, to keep up the abnormal opening. Often it is unnecessary to keep the sound in permanent place, as the fistula may cicatrize as soon as the urethra offers no further obstacle to the flow of urine. Unfortunately, it is not always thus, some of these fistulas remain, though the canal may be perfectly free. This persistence may be owing to the presence of gravel in the fistulous tract, or else to more or less separation of adjacent and subjacent tissues. In such cases, extract the gravel, and cause the adhesion of the separated parts. These are the evident indications to be fulfilled. I have succeeded in curing multiple fistulas by the buttonhole treatment of Syme, being careful to incise each sinus. But this is a grave operation, and, moreover, there are some cases kept up by neither gravel nor by decollement, but by the continuous passage of a few drops of urine. Cauterization of the urinal orifice will not avail, for the urine is introduced from above, and nothing can impede its effect upon cicitrization; or, if the lower orifice should close, the effusion will soon reopen it. What, then, must we do to cause the closure of these fistulas? What, then, must we do to cause cicatrization. Modify the internal orifice. Cauterization of this orifice furnishes the most proper means to bring about this result, by freshening the parts, bringing about a healthy granulation, and also producing a temporary obliteration of the opening by the swelling of its borders and by the eschar which protects the farther parts of the urethra and permits them to rejoin.

Such are the ideas that induced me to apply, by means of the endoscope, the nitrate of silver to the urethral orifice of stubborn fistulas. The process for doing this is most simple:—

Having, by proper treatment, caused the disappearance of all obstacles to the flow of urine and the passage of the sound, by the aid of the endoscope, we examine the urethra from back forwards, until we find the opening of the fistula. This is sometimes difficult to do, because its edges may touch, or it may be located in the midst of an ulceration, but it may generally be discovered by means of a small violet red spot or one of the color of wine dregs. Often, a stylet or bougie can be introduced into the fistula and will show itself at the end of the endoscope, thus indicating its urethral opening.

In a case now in my service, traumatic in its origin, the orifice was recognized by means of a fungous vegetation, similar to those often found at the external opening of urinary fistulas. Two cauterizations destroyed this vegetation and laid bare the opening. The patient is still under treatment. In this operation, eschars, so far from being feared, must be hoped for, they are necessary. A small pencil of solid nitrate of silver, placed in an appropriate porte, should be used; in the absence of a proper instrument, melt the nitrate upon the end of the endoscopic stylet.

When the orifice of the fistula is discovered, nitrate of silver is to be applied, care being taken not to touch neighboring parts; it is to be left a short time, and efforts made to introduce it into the orifice. When it is thought that sufficient action has accrued, the caustic is to be withdrawn, and, to prevent excessive action upon the neighboring parts, cotton impregnated with marine salt should be introduced, to transform the nitrate into an inoffensive chloride.

After the operation, there is but little pain, rather more, perhaps, than after the application of a solution of nitrate of silver, but still very bearable. The day following the cauterization, the passage of urine through the fistula is generally diminished and sometimes completely checked; yet, for all that, it must not be supposed that the patient is well, though such effects, to my great surprise, I have more than once experienced. The swelling which succeeds the action of the caustic soon subsides, the eschar falls, and the urine recommences to pass. Thus it is necessary to wait for some days so as to give the work of reparation time to set up the recommencement. The urine gradually ceases to pass through the fistulous opening, which ends by drying up (*se tarir*), and the cure is complete.

The following case will show the march of the cure:—

*Slight Contraction of the Bulbous Portion—Laceration of the Canal behind the Stricture—Infiltration of Urine—Incision —Fistula—Cauterization of the Urethral Wound—Cure.*

D., aged 35, employed upon the railway, married.

His general health good. Before his marriage he had a light blennorrhagia, which lasted fifteen days, and then disappeared, he says, without taking on the chronic form. During the past five or six months, however, he has experienced occasionally, and especially when heated, difficulty in urinating.

On the 6th of July, 1862, while transporting baggage to the cars, a heavy trunk fell upon his left ilio-pectional eminence. He felt considerable pain therefrom, but still continued his work. The next day, the pain continued, and he perceived a small tumor formed upon the course of the left spermatic cord; gradually the surrounding parts became swollen, and micturition difficult and painful. Still this did not impede his work for seven days yet. At length he decided to keep his bed, and called in medical aid. Hip-baths and leeches were ordered; then he came to the hospital.

On his entrance, he could not possibly urinate; he suffered much from this cause and from the tumor, now the size of a hen's egg, vertically elongated, in front of the left inguinal region, now hard and not fluctuating. Catheterism presents no great difficulty and gives issue to much urine, greatly to the relief of the patient.

The marrow, the scrotum, the penis, the left groin, the perineum are the seat of great œdematous swelling. The patient is most anxious; there is pain in the parts; and some febrile symptoms. Four incisions are made, two in the perineum, one in the left scrotum, and one in the groin; thence, came much pus and serosity; the pus without the smell of urine. Cataplasm.

The parts disgorge in proportion to the discharge from the incisions. A few days after, the pus takes on a urinous smell. A sound is left in the bladder. His general condition is excellent; no chills, no fever, and a good appetite.

In a fortnight, two of the incisions are cicatrized; the third requires two months to close; the fourth, situated at the left antero-superior part of the scrotum, remains fistulous, and, when the patient urinates, a portion of the liquid passes through it. He also perceives it in the cataplasm used on the wound. Bougies of Béniqué are passed, from No. 34 to No. 51.

*9th Oct.* Endoscopic examination, A violet red spot, easy to bleed, is found in the inferior half-circumference of the canal; this spot seems to surround the internal orifice. It is cauterized with a concentrated solution of nitrate of silver, applied by means of a cotton tampon passed through the sound of the endoscope. The permanent sound is withdrawn. The urine, the first time, passes with difficulty and in a scattering stream, but the ensuing day it becomes perfectly natural. The urinary discharge through the wound still continues.

*16th.* Second cauterization, by means of the solid stick of lunar caustic passed through the sound of the endoscope. During the day, there is a slight hemorrhage; the micturition is properly made about every hour. The discharge by the fistula diminishes in quantity.

*23d—30th.* The fistulous opening in the canal is again touched with the stick of silver. Metal bougies are passed almost every day. The improvement continues, and, on the *4th Nov.*, the patient is discharged, perfectly cured, the urethral fistula cicatrized, and the urine passes in a full and copious jet.

### FIFTH LECTURE.

### *The Prostate.—The Bladder.—Calculi.*

GENTLEMEN:—We have now finished the study of the application of the endoscope to the diseases of the urethra; but you doubtless recollect that, in speaking to you of urethral granulations, I omitted those affecting the prostate, deferring their description to a later period. With these, we will commence our lesson to-day.

Ulcerations of the urethra, whether granular, herpetic, or arthritic, present the same characters in the prostatic as in other portions of the canal, only they more readily become fungous, and, consequently, it more often happens their character is masked, and thus it may be more difficult to distinguish them. In such cases, diagnosis must be based upon the progress and other symptoms. We must, however, remark that, most frequently, the endoscope will enable us to distinguish between these ulcerations.

Granular ulcerations in the bulbous and membranous region are, as we have seen, the most frequent cause of stricture, in consequence of the organic changes they cause, and which we have fully examined. In the herpetic region, owing to the different anatomical arrangement, retractions do not occur, as I have before said, but the congestion, the irritation kept up by the granulations, may end in a chronic engorgement of the gland. It might be said of the prostate as it has been of the uterus, that it is not the ulceration which brings about the engorgement, but the engorgement which supports the ulceration. It is certain, that in the prostate, as in the uterine neck, there are ulcerations kept up by organic chronic engorgement; but study them well, and you will find that these ulcerations, which increase and decrease, disappear, even under the influence of treatment, and reappear without apparent cause upon the engorged organ, on ulcerations of an herpetic character, which can be as readily distinguished here as in the anterior parts of the urethra, though they possess less mobility. As to the true granular ulcerations, in the prostate as in the bulb, the uterine neck, or the conjunctiva, once cured, they do not reappear without the reapplication of the exciting cause.

There is no particular treatment for this affection, but if it should be necessary to employ special means, it should be to combat certain complications, such as inflammatory symptoms of the parenchyma of the gland, or of certain accidents caused by a swelling of this organ. For the rest, the endoscope gives us the means of attacking, efficaciously, ulceration which is no longer more difficult to cure than in any other region.

We have seen that the chronic inflammatory stricture heals spontaneously at the same time as the granulations which maintain it, but when it arrives at a certain point, whilst the swelling is more or less inflammatory, and the induration is established, it is obstinate in its course, and is the same as engorgement of the prostate.

I have often seen the prostate take on its volume and its functions reëstablish themselves in their normal state, in proportion as the granulations yield to to treatment. Thus, in the prostate as in the rest of the canal, the granulations have the same character, the same progress, and yield to the same treatment; they act through a mechanism analogous to the mucous and the subjacent tissues. But here the analogies end, for the effects produced are not the same. So long as the contraction is almost exclusively due to the granulations, the engorgement of the prostate is well known to arise from other causes. They appear more frequently to arise from arthritic and herpetic diathesis, from scrofula than from hygienic influences whose actions are universally known. Of all the causes resulting from affections differing in their nature, but the symptomatic expression of which is somewhat identical, it is almost impossible to-day to arrive at a diagnosis with anything like certainty. We only hope that, with the era of endoscopic observations, we shall be able to elucidate the subject now so obscure to science. Already, the endoscope has permitted us to perceive the engorgements due to granulations. In the scrofulous engorgements with ulcerations, it gives also some positive characteristics.

In effect, when these ulcerations remain for some time, they go beyond the limits of the mucous, and then, in place of a superficial denudation, the endoscope reveals to us a true ulcer, deep and ragged, presenting, in a word, the appearance of a strumous ulcer.

We had, at the commencement of the year, in our ward, a patient afflicted with strumous ulceration and tubercles of the prostate, with urinary fistula of the same nature, upon which these characters grew more and more distinct in proportion as the lesion progressed. This man left us, thinking he could find elsewhere a cure, which he despaired of obtaining with us. In the case of this gentleman, you are well aware that the therapeutic treatment is very limited, but, however, it will be in our power, thanks to the endoscope, to convey to these deep-seated lesions the same topical treatment which we have known by expesience to act favorably upon lesions of the same nature in exterior organs.

A consequence attending fixed ulcerations in the prostatic regions of the urethra, which oftentimes injures the patient, are seminal losses. It happens oftentimes that they constitute a constant symptom, or even become habitual, in fact, they return with sufficient frequence to merit special attention; but so much the more, without having exaggerated the importance which we have attributed to them, we can but arrive at the conclusion that they may become exceedingly grave. But, whilst they derive their existence from the ulcerations, they seem to disappear of themselves, or by simple means after the recovery.

This lesion may return under two forms, one, which can be attributed to the irritation of the orifices of the ejaculatory canals, accompanied by lively sensations, and often by profound pains of the perineum; the other, which appears atonic, comes on with little sensation, sometimes in sleep, without the knowledge of the patient, and without awaking him; whilst he is awake, if the patient wishes to accomplish his virile functions, the least excitation will bring on the emission, and, as M. Trousseau has said in his lectures, it can but result in a sort of impotence, to which we ought to give the name of "spermatic incontinence."

In the one or in the other case, but oftentimes in the first, the semen which the patient loses is often mixed with blood, which proves the existence of the ulceration. This symptom may appear equally in all ulcerations, but above all in granular ulcerations; the losses which accompany tuberculous ulcerations appear more frequently from the atonic form; those which proceed from herpetic ulcerations are less obstinate than the others.

Apropos to this subject, another very similar symptom presents itself, which very much surprises and astonishes him who experiences it: the patient, in his sleep, experiences all the sensations of a nocturnal pollution, he awakes, and, with astonishment, perceives that no liquid has been emitted.

The idea which naturally presents itself, is that of a deviation of the ejaculatory canals which directs the sperm towards the bladder; but without taking into account the disorders which it is necessary to admit must arise from a displacement or deviation of the ejaculatory canals, the patients which I have had under my observation formerly, from normal ejaculations and elsewhere, the most minute microscopical researches do not show any trace of spermatozoa in the urine. There had been then, in the few cases that I have observed, a false sensation, a sensation without an emission.

This phenomenon is not, like the rest, a certain index of a prostatic affection, for I have seen healthy men, perfectly well, from this country in particular, who had experienced it; only in these cases it did not return or renew itself, as in the case of the patients.

A symptom oftentimes more rare, and which I have seen alike upon patients affected with granular ulcerations of the prostate, is a sensation analogous to that which accompanies coition, a sensation which becomes, for the moment, painful, or, as a patient said to me, "a voluptuous pain," which endures without interruption during the entire coitus, and oftentimes for weeks, and horribly fatigues those who endure it. In the two patients who presented this symptom in the highest degree, it was accompanied by a pain sufficiently sharp that the patient located it at a certain point in the neck of the bladder. These two cases prove, beyond doubt, some nervous affections which have a great resemblance to hysteria. The one most gravely affected, of my two patients, had become a melancholy hypochondriac and had given serious uneasiness to his friends, and distinguished physicians had attributed the entire affection to a nervous disease.

In these patients, the prostatic region presented a granular ulceration; one of them, cured of granulations, was at the same time embarrassed with pains and all the other symptoms which attend an old catarrh of the bladder. The other patient, cured of his granular ulceration, preserved his fixed pain, which he said had its seat at the left of the opening of the urethra into the bladder, at a point where the canal is continued with the vesical cavity. He entreated me always to pay particular attention to this point, assuring me that I should find there the cause of his difficulty. In fact, when I commenced, after having emptied the bladder, to examine minutely the border of the orifice of the urethra, I discovered to the left a small, well-marked, red ulceration as large as the head of a large pin, which resembled a small fissure. I touched it with a small crayon of nitrate of silver, the pains disappeared almost immediately, obscured by the pain of the cauterization, and did not reappear; the patient became perfectly calm. The patient returned twice, at somewhat long intervals, for his pains had revived, and the same treatment allayed them. For many months, the patient appeared to me to be cured. I met him from time to time, he did not speak of his difficulty, but from his exterior or general appearance, I am inclined to think that this melancholy hypochondriac is completely cured, as also the lesion which produced it. I do not know in what class of diseases to place the cases of these two men.

We have seen seminal losses caused by the ulceration of the prostatic region of the urethra, but it is not necessary to believe that they always arise from this cause, for they may originate from diseases of different nature and often remote or distinct from the genital organs.

In the enumeration which we have made of diseases of the prostatic portion of the urethra, we have not spoken of cancer, because it determines beyond doubt some symptoms which report themselves from the bladder, and which cannot be relieved or cured without attention to this organ. We will, therefore, return to this organ at once and speak of affections of the bladder, and, above all, of those which pertain to the neck of this organ. We will now occupy ourselves, gentlemen, with an endoscopic exploration of the bladder, and, as we did with the urethra, we will commence by the study of its healthy condition.

In order to examine the bladder with an open sound, like those which we used for the urethra, it will be necessary to clear it, for if the extremity of the sound becomes clogged with mucus it will be impossible to proceed into the cavity of the organ, in order to explore all the points. It is necessary then that the bladder be full at the moment of exploration, and, in consequence, we must employ a sound which will stop the flow of urine and, at the same time, allow the rays of light to pass in.

This sound presents the same form as the urethral, but it has not a lateral opening, and terminates by a little prolongment around the end, which separates itself at an obtuse angle; in a manner, it resembles, in its general form, the prostatic sound of M. Mercier, the introduction of which is so easy, as we have said, especially when there is a swelling of the prostate. At the angle of reunion of the two branches, the sound is pierced in the axis of the long branch by an oblique opening, closed by a little plate of glass with faucets, plain and parallel, carefully and solidly set in with mastic. It is this glass which retains the liquid and allows the light to pass through; it should be oblique, because if it was perpendicular to the axis of the tube, it would be less clear before the vesical cavity than the interior of the instrument, it would make a mirror and reflect the illuminated points situated in front of it, and prevent from distinguishing points situated beyond it. Thus, also, at night, when we direct a strong light towards the outside of a window, we see in the glass, as in a mirror, the inside of the apartment, whilst the exterior appears entirely obscure.

The extremity of the small branch of the sound can be screwed on, so as to facilitate the cleaning of the interior face of the glass, this, however, is rarely necessary. We must be careful that the small addition be always well screwed on, so as not to let the liquid pass through it, for the smallest drop in the interior of the sound will interfere with the rays of light. You will easily understand, gentlemen, that if the bladder should contain a turbid liquid it will be impossible to distinguish anything of its interior structure, whilst it is diseased it is rarely that the urine is not made turbid by mucus, pus, or blood, and often by these three liquids mixed in various proportions, it will then be necessary to empty the bladder by the aid of an ordinary sound, which will serve also for the purpose of injections, so that it may be washed out.

When the water of the injections becomes very clear, then we may withdraw the ordinary sound and replace it with the "sonde a vern" which we have just described. Upon this sound we shall place the apparatus, which we have described in our first lesson, and shall then commence to examine. In an open or clear bladder, there is nothing to constrain the movements of the instrument, we can thus explore the smallest part of the internal surface of this organ, even to the border of the prostate, the vesical triangle, and the superior and inferior fundus. The anterior face escapes the examination; I have essayed to make this accessible, by means of a mirror placed at the extremity of the long portion of the sound, in front of the lateral opening, but the clearness not being sufficient, the field of vision is considerably curtailed, and, in fact, the result amounts to nothing. I have not yet, however, renounced the hope of being successful, but, for the present, we must content ourselves with examiuing that portion which is accessible to us, then, by a happy coincidence, this portion is precisely the one which is most often diseased, and that which escapes our examination very seldom offers anything of interest.

We shall now learn, through the endoscope, the physiological condition of the bladder. The mucous membrane of the bladder presents a healthy and smooth surface, and a color analogous to that of the urethra, but ordinarily a little more pale, it is of a yellowish white, slightly rose tinted.

In certain subjects it presents, here and there, a number of superficial vessels, very minute, which are seen to appear at different points, and ramifying themselves, take very tortuous courses. Very often, these vessels are not seen in a bladder well filled, and one cannot perceive in the points which present themselves in the field of the instrument, that the surface is smooth and the color uniform. The only particularity that we have noted is, that we can reconnoitre, whilst it exists, the transverse angle or point which limits the "trigonus vesicale" from behind, and extends between the orifices of the two ureters. In regard to the size of these orifices, I have not cared to speak to you, because it is unnecessary.

In order to examine the urethral orifice, it is necessary to withdraw the sound as far as where the glass commences to reënter the urethra, then we commence to perceive the inferior part of the visual field, the border of the prostate, under the form of a small crossing from the superior concavity. This part is habitually more red than the vesical mucous membrane, and as the border which it forms intercepts the rays of light, the surface of the bladder oftentimes appears of a deeper color.

If we carry the extremity of the sound to the right or left, we can follow the contour of the urethral orifice; but, as in a moment, one loses the borders of this orifice, at the same time that it passes away, in place of appearing circular, its contour forms a crook, elongated transversely. But the state of health as we pass to the disease, commencing with the case which approaches least to a normal state, we shall find the borders of the organ anæmic and the congestion characteristic, the one by its pallor, the other by the redness of the membrane, in which the vessels become oftentimes more apparent. I have encountered the anæmic pallor in chlorotics or very feeble patients, and oftentimes in some cases of neuralgia of the bladder in chlorotic or debilitated subjects, the dilatation of the capillary vessels, with or without redness of the mucous membrane; sometimes, likewise, they detach themselves upon the healthy membrane, and oftentimes it is the only lesion which the endoscope shows in patients affected with hematuria, without calculi or organic affections. In this case, we often diagnose varices of the neck. They perhaps exist sometimes, but more frequent from my observations, already sufficiently numerous if one can suppose that the blood be furnished by the varices. There are some capillary varices which extend themselves over the entire membrane, or at least a great part of it.

Without these vascular dilatations in the case of hematuria, we encounter, sometimes, some ecchymoses of the membrane, sometimes considerably large and irregular, sometimes small and lenticular. I have observed, in two cases of scorbutic hematuria, that the color somewhat resembled a violet tint. I shall reapproach these light affections, grave without doubt, but more so in appearance than reality, the diagnosis of which would be difficult without the aid of the sight.

We shall meet with subjects which are taken, it may be in full health, or it may be in the course of some light affection or habit of the urinary functions, with violent pains of the neck of the bladder, with vesical tenesmus, hematuria, retention of urine, more or less complete; these symptoms, after a variable duration, subside rapidly, sometimes suddenly, but often they return at intervals more or less in length.

With like symptoms, and in spite of their complete subsidence, for sometimes there is no rest between the attacks, it is difficult not to believe that probably a grave and organic disease of the bladder exists, then whilst we cannot diagnose any tumor by catheterism. I have often known given, and I myself have held, in similar cases, an unlucky diagnosis. It is difficult to avoid it with the ordinary means of diagnosis; but apply the endoscope, examine the bladder with care, and you will find an exaggerated development of the capillary vessels of the mucous membrane.

Whilst I have had occasion to observe some cases of this kind, it was upon patients subject to attacks of gout and rheumatism, and some vesical affections so closely resemble arthritic affections that I have been almost obliged to attribute them to the same cause, and as I regard this abnormal dilatation of the capillary vessels as one of the effects of arthritis, for the same reason that the capillary varices of the inferior members and of some other parts of the tegumentary system.

After the congestion of the mucous membrane, we arrive naturally at its inflammation. Acute cystitis cannot exist without danger or without pain, it cannot result in any advantage to the sick to practice an exploration by the aid of the endoscope, but if it be only chronic cystitis or sub-acute, and of that which accompanies the presence of calculi in the bladder, then it is practicable to examine without danger, and with utility, and to make an exact diagnosis of the lesion. In the first degree, we find a general redness with development of the vessels, which are oftentimes more numerous but less dilated than in the affections of which we have spoken.

In a degree more advanced, the redness of the mucus is more intense, and we cease to distinguish the vessels which are confounded in the general well-grounded tint. So that, in certain chronic cystitis, for a long time there is a general ramollissement of the mucous membrane which has lost its epithelium, and the surface of which has become uneven, rugged; in the autopsy we find it black, in consequence of the alteration which, after sudden death takes place in the gorged bloodvessels. In this case, it is often difficult to separate the bladder from the pus that it contains, and of which the thickest part rests adherent to the ulcerated surfaces, but if you proceed to fill the organ with limpid water, you will find the membrane of a deep red in places, and presenting the same rugged aspect that we see in

